# Seizure relapse in new onset epilepsy: It is not always drug resistance

**DOI:** 10.1002/epi.70298

**Published:** 2026-06-11

**Authors:** Cecilia Catania, Eric Menetre, Aikaterini Fitsiori, Felix Tobias Kurz, Gabriela Moreno Legast, Serge Vulliemoz, Mathieu Genoud, Pierre Mégevand, Roland Wiest, Fabienne Picard, Sandor Beniczky, Margitta Seeck

**Affiliations:** ^1^ Department of Clinical Neurosciences, EEG & Epilepsy Unit University Hospitals of Geneva Geneva Switzerland; ^2^ Diagnostic and Interventional Neuroradiology Unit Berne Switzerland; ^3^ CIBM Center for Biomedical Imaging Lausanne Switzerland; ^4^ Emergency Department University Hospitals of Geneva Geneva Switzerland; ^5^ Diagnostic and Interventional Neuroradiology, University Hospital Berne Switzerland; ^6^ Department of Clinical Neurophysiology Copenhagen University Hospital Copenhagen Denmark; ^7^ Department of Clinical Neurophysiology Danish Epilepsy Centre Dianalund Denmark

**Keywords:** acute symptomatic seizure, adherence, adults, antiepileptic treatment, antiseizure medication, compliance, EEG, epilepsy surgery, first seizure, functional/dissociative seizures, nonepileptic seizures, pharmacoresistance

## Abstract

**Objective:**

Seizure recurrence in new onset epilepsy (NOE) can result from various factors. Although drug ineffectiveness is frequently investigated, other causes—such as nonadherence, inadequate treatment, nonepileptic events (e.g., functional/dissociative), or acute symptomatic seizures—also impact patient outcomes. This retrospective, longitudinal single‐center study evaluated relapse causes over a 5‐year period in a cohort of NOE patients.

**Methods:**

We reviewed the NOE registry of Geneva University Hospital, including adult patients diagnosed between 2005 and 2019. Of 740 consecutive patients, 330 with more than 5 years of follow‐up were included in the analysis. Relapses after antiseizure medication (ASM) initiation were classified into seven categories: poor adherence, inadequate treatment, ineffective treatment, acute symptomatic seizures, nonepileptic seizures, multiple, and unknown causes. Mixed‐effects logistic regression and post hoc analyses were used to assess temporal trends.

**Results:**

A total of 202 of 330 patients (61%) experienced at least one relapse; 181 occurred after ASM initiation. Of these, 32% (57/181) were due to treatment ineffectiveness, whereas 68% were attributable to other causes. Inadequate treatment was the most frequent cause in year 1 (37%) but declined to 10% by year 5 (*p* < .001). Poor adherence remained stable (27%–38%). The proportion of drug ineffectiveness as a cause of relapse increased over time (22% in year 1 to 41% in year 5). Drug‐resistant epilepsy (DRE) was diagnosed in 7% of the entire cohort. Generalized tonic–clonic (GTC) seizures at relapse were associated with poor adherence, whereas non‐GTC seizures were linked to ineffectiveness (*p* < .001).

**Significance:**

In our cohort, most early relapses in NOE were due to modifiable factors. Systematic identification of relapse causes can prevent misclassification of DRE and reduce unnecessary treatment escalation. The high proportion of patients lost to follow‐up is a relevant limitation of this study; however, a substantial selection bias is unlikely.

**Key points**

- Seizure relapse rates in NOE decrease significantly over time after diagnosis.
- Relapses due to inadequate treatment predominate in year 1 and decline; poor treatment adherence and drug ineffectiveness remain stable over time.
- The proportion of patients meeting criteria for drug‐resistant epilepsy increases over time.
- Generalized tonic–clonic seizure relapses are primarily associated with poor treatment adherence.

## 1 INTRODUCTION

Epileptic seizures occur in approximately 8%–10% of the population.^1^ Most studies reported 40%–50% relapses at 2 years,^2^, ^3^, ^4^ although lower numbers (27%) have been also found in older studies.^5^ Whereas some research has investigated specific causes of relapse, such as poor adherence,^6^ a comprehensive understanding of the distribution of different causes of relapse in the first years after the onset of epilepsy remains limited.

First‐time seizures can be divided into four main categories: symptoms of new onset epilepsy (NOE), unprovoked epileptic seizures in patients not fulfilling the criteria for epilepsy diagnosis, acute symptomatic seizures (ASyS; formerly also called “provoked seizures”), and manifestations of nonepileptic events (such as syncope or functional/dissociative seizures). It is known that the recurrence rate depends critically on correct diagnosis and appropriate treatment. However, there are few studies on the precise causes of recurrence after the first seizure. In patients with NOE, a seizure recurrence may be due to ineffective treatment, but also to poor adherence to therapy, inadequate treatment, low drug levels, ASyS, or nonepileptic events. Furthermore, not all recurrences in a single patient have the same cause. Investigating relapses is important, because they carry a similar risk of morbidity and mortality as the first attack and can be mistaken for ineffective treatment.

In this study, based on our registry of consecutive patients with NOE diagnosis in the Canton of Geneva, we investigated the causes of each relapse, their changes over time, and the emergence of drug resistance to one or two medications within the first 5 years after epilepsy diagnosis. All patients underwent comprehensive examinations from the time they were enrolled in our registry, thus providing a unique opportunity to explore the true prevalence of drug resistance.

## 2 MATERIALS AND METHODS

### 2.1 Patient selection

This retrospective longitudinal study was conducted at the University Hospitals of Geneva, based on a registry of patients aged >16 years who were admitted to the emergency department (ED) or the adult neurology department with suspected first epileptic seizure between 2005 and 2019 who were subsequently diagnosed with NOE. University Hospitals of Geneva are the only hospitals in the canton providing 24‐h, 7 days per week on call neurological services, and therefore they receive almost all patients who seek medical attention for first‐time events involving abnormal movements, transient sensory phenomena, or loss of consciousness.^7^ The hospital's catchment area comprises approximately 530 000 inhabitants.

Patients were enrolled according to the following inclusion criteria:

1. Patients with a first epileptic seizure (index event), admitted to the ED or adult neurology department.
2. Diagnosis of NOE (based on the International League Against Epilepsy [ILAE] criteria): (1) at least two unprovoked seizures occurring more than 24 h apart, (2) one unprovoked seizure with a ≥60% probability of further seizures over the next 10 years, or (3) diagnosis of an epilepsy syndrome.^8^, ^9^ In clinical practice, NOE may be diagnosed and antiseizure treatment initiated after a single unprovoked seizure when epileptiform abnormalities are detected on electroencephalography (EEG) or an epileptogenic lesion is identified on brain imaging.^10^, ^11^, ^12^
3. Availability of at least 5 years of follow‐up data.

Exclusion criteria were:

1. Age less than 16 years.
2. Index event classified as clearly nonepileptic or as ASyS at the first event,^13^ or single unprovoked seizure not meeting the criteria for NOE.

### 2.2 Diagnostic workup

Following the initial event, the attending neurologist obtained a comprehensive medical history, focusing on the symptoms, similar incidents, and potential triggers. When available, witness statements were also obtained to supplement the clinical assessment. All patients underwent brain imaging (computed tomography [CT] or magnetic resonance imaging [MRI]) after the index event, most (288/330, 87.3%) within the first week. A routine 20–30‐min EEG was performed using 19 or 25 electrodes, in accordance with International Federation of Clinical Neurophysiology recommendations.^14^ If these baseline examinations were unremarkable, a long‐term EEG (LT‐EEG) with a nightly recording using 21 or 25 electrodes and an average duration of approximately 18 h was performed either immediately after the index event or in the following weeks, depending on logistical feasibility and patient agenda, often during a short hospital stay. Both routine and LT‐EEG recordings were reviewed for interictal and ictal epileptiform abnormalities. Standard electrocardiography and basic laboratory tests were also performed to investigate other possible causes outside the spectrum of epilepsy diagnosis.

If the clinical evaluation confirmed the suspicion of NOE, antiseizure medication (ASM) treatment was initiated. In cases of alternative diagnoses, patients were referred to the appropriate specialty. If the diagnosis remained unclear, patients were followed to review the diagnosis over time and consider clinical findings.

Patients diagnosed with epilepsy underwent regular follow‐up examinations, including checkups at least 3 and 6–9 months after the index event, followed by annual visits. Medical records were reviewed for up to 5 years after the index event. Most patients were managed by our epileptology team, whereas a small number were monitored by external neurologists who did the necessary workup after each relapse and provided updates on their condition. Clinical data were reviewed for accuracy by two board‐certified neurologists (M.S., C.C.); any discrepancies were resolved through consensus. The study was approved by the relevant ethics committee, and all participants provided informed consent.

### 2.3 Definitions

Focal epilepsy was diagnosed when semiology, neuroimaging, or EEG (routine or LT‐EEG) findings indicated focal onset. Structural epilepsy was defined as seizures associated with identifiable epileptogenic brain lesions likely to explain the patient's seizures. Idiopathic/genetic generalized epilepsy included syndromes characterized by typical seizure types and EEG patterns, in line with the ILAE classification.^15^

Relapses were defined as seizure events either identical to the index event or presenting with semiology congruent with the patient's epilepsy type. Relapses were systematically identified either during follow‐up consultations or through ED visits.

In the case of a relapse, the circumstances and symptoms were examined and classified. Possible triggering or contributing factors, including potential pharmacological interactions, were systematically evaluated. Any recurrence of seizures prior to the start of treatment was also documented. A delay in initiating treatment was observed if necessary diagnostic tests could not be performed promptly, initial results were inconclusive, or the patient sought a second opinion.

Relapse causes after treatment initiation were categorized as follows:

1. Inadequate treatment: Included patients with any of the following conditions: (1) suboptimal dosing due to ongoing titration, (2) documented and known drug–drug interactions potentially reducing ASM efficacy, (3) prescription of ASM not recommended for the specific epilepsy type, or (4) prescribed dosage below 75% of the defined daily dose.^16^
2. Poor adherence: Documented history of nonadherence temporally associated with the relapse and/or low ASM blood levels despite an adequate prescribed dosage.
3. ASyS: Relapses occurring in close temporal relationship with acute central nervous system insult, that is, due to metabolic, toxic, structural, infectious causes, or due to inflammation.^17^
4. Functional/dissociative seizures (FDS): Abrupt, paroxysmal changes in behavior, consciousness, or motor activity resembling epileptic seizures, not caused by abnormal electrical activity in the brain.^18^ The diagnosis was considered based on clinical assessment with evidence of a different semiology, including psychiatric consultation and normal overnight EEG.^19^ We then reviewed the initial event and excluded those patients if we concluded that the first seizure was misdiagnosed and was also an FDS. Thus, only patients with a first epileptic seizure who subsequently experienced FDS were included in the present analysis.
5. Treatment ineffectiveness: Persistence of seizures despite adequate ASM, appropriate dosages, and confirmed good adherence, demonstrated by at least two therapeutic ASM blood levels within the recommended range and/or reliable external confirmation (e.g., from caregivers) that treatment had not been discontinued.
6. Multiple causes: Applied when a patient experienced seizure recurrence in the same year attributable to more than one identifiable cause.
7. Unknown cause: Assigned when relevant information regarding the clinical context of seizure recurrence was missing or unavailable.

Drug‐resistant epilepsy (DRE) was defined according to standard criteria as the failure of adequate treatment attempts with two tolerated, appropriately selected, and correctly administered ASMs (both as monotherapy and in combination), beginning after the first clinical event. Patients who did not achieve seizure freedom but underwent only one adequate ASM attempt were classified as having undefined drug responsiveness.^20^

### 2.4 Statistical analysis

For each patient, demographic and clinical variables, including age, sex, epilepsy type, presence of progressive or structural epileptogenic lesions, history of prior uninvestigated seizures, prescribed ASM, and type of relapse, were retrieved from the patients' chart. Comparisons between patients with and without relapse were performed. Data analyses were performed using R software (V.4.3.2).

The occurrence of different relapse causes over time was assessed using a mixed‐effects logistic regression model. Post hoc analyses assessed changes in the distribution of relapse causes over the 5‐year follow‐up, as well as associations between specific causes and prior clinical events. Associations with clinical variables were examined using logistic regression.

Changes in the proportion of subjects meeting the criteria for DRE during follow‐up were evaluated using Cochrane *Q* test, whereas year‐to‐year comparisons were assessed by McNemar test. A generalized linear mixed model with a binomial distribution was used to evaluate the association between seizure type (generalized tonic–clonic seizures [GTCS] vs. non‐GTCS) and relapse cause over time. Interaction terms were included to assess whether seizure type distribution varied according to the cause of relapse throughout the follow‐up period.

## 3 RESULTS

### 3.1 Patient characteristics and relapse rate

From a total of 1000 patients in the registry, we identified a total of 740 consecutive patients diagnosed with NOE between 2005 and 2019. Of these, 330 had at least 5 years of follow‐up. The remaining 410 moved away from the canton of Geneva and could not be followed up further or were managed by private neurologists, without in‐depth workup of the underlying cause. The mean age at epilepsy onset was 49.3 years (±20 SD, range = 16–97, median = 49.4), and 42% of the subjects were women (Table 1). A history of probable, unexplained previous seizures was noted in 21% of the patients. The majority of patients (309/330) underwent brain MRI, whereas 21 had only a head CT. This was due to the presence of non‐MRI‐compatible metallic devices (e.g., pacemakers or metallic clips; 3/330), the presence of a clear and already known focal cause (e.g., ischemic or hemorrhagic stroke, subdural hematoma; 15/330), or patients with cognitive impairment requiring general anesthesia to undergo MRI (3/330).

**TABLE 1:** Patient characteristics.

Characteristic	*n* (% of total)
Total patients, *N*	330
M	192 (58)
F	138 (42)
Mean age, years (SD)	49.3 (20.8)
History of possible untreated seizures	
Yes	69 (21)
No	128 (39)
Epilepsy types	
Focal structural epilepsy	247 (75)
Focal nonstructural epilepsy	49 (15)
Genetic [idiopathic] generalized epilepsy	34 (10)
Types of brain lesion	
Vascular	101 (41)
Brain tumor	49 (20)
Atrophy	21 (9)
Trauma	25 (10)
Developmental malformation	15 (6)
Infectious/inflammatory	13 (5)
Gliosis [postsurgical, HS, unspecified]	19 (7)
Other	4 (2)
Medication	
Levetiracetam	138 (42)
Valproate	101 (31)
Lamotrigine	53 (16)
Carbamazepine	14 (4)
Other	24 (7)
Relapse	202 (61)
Only before ASM introduction	21 (6)
After ASM introduction	181 (55)

Abbreviations: ASM, antiseizure medication; F, female; HS, hippocampal sclerosis; M, male.

Following diagnostic workup, ASM was initiated. Most patients (61%) began treatment on the same day as the index event. Levetiracetam (42%) was prescribed most frequently, followed by valproate (31%) and lamotrigine (16%). No significant differences in relapse frequency were observed between the various ASMs.

Relapses occurred in 202 of the 330 patients with 5 years of follow‐up (61%). In 21 patients (6.4%), a second seizure only occurred before treatment initiation. Therefore, in the analysis of relapse causes, we considered only the 181 patients who experienced at least one seizure after the introduction of ASM for the current analysis.

Detailed data on seizure frequency by seizure type and relapse etiology are provided in Tables S1–S4. Forty‐one patients experienced only one relapse (23%), 44% of which were due to suboptimal treatment and 32% to poor adherence. Twenty‐three patients had two relapses (13%), and the remaining 117 (64%) had three or more.

**FIGURE 1:**
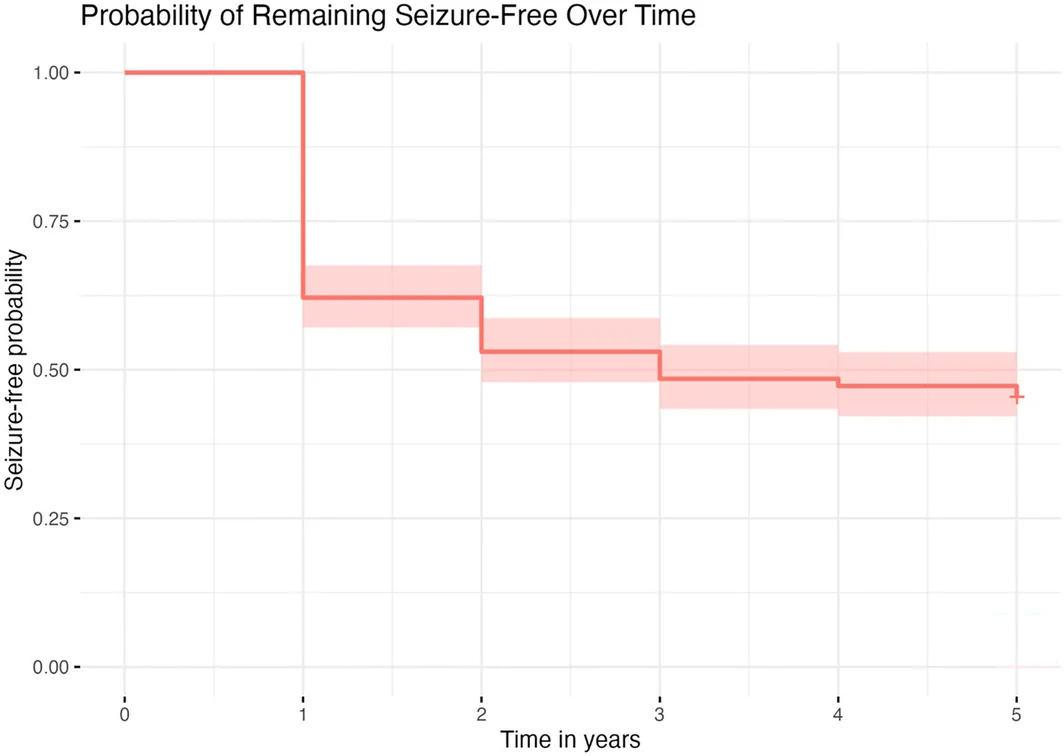
Kaplan–Meier curve of the probability of patients with new onset epilepsy diagnosed after a first seizure of remaining seizure‐free. Please note the sharp decline after the first year.

The relapse rate decreased progressively: 125 (38% of the entire cohort) patients in the first year, 102 (31%) in the second year, 88 (27%) in the third year, 74 (22%) in the fourth year, and 62 (18%) in the fifth year (*p* < .001; Figure 1).

### 3.2 Causes of relapse during follow‐up

**FIGURE 2:**
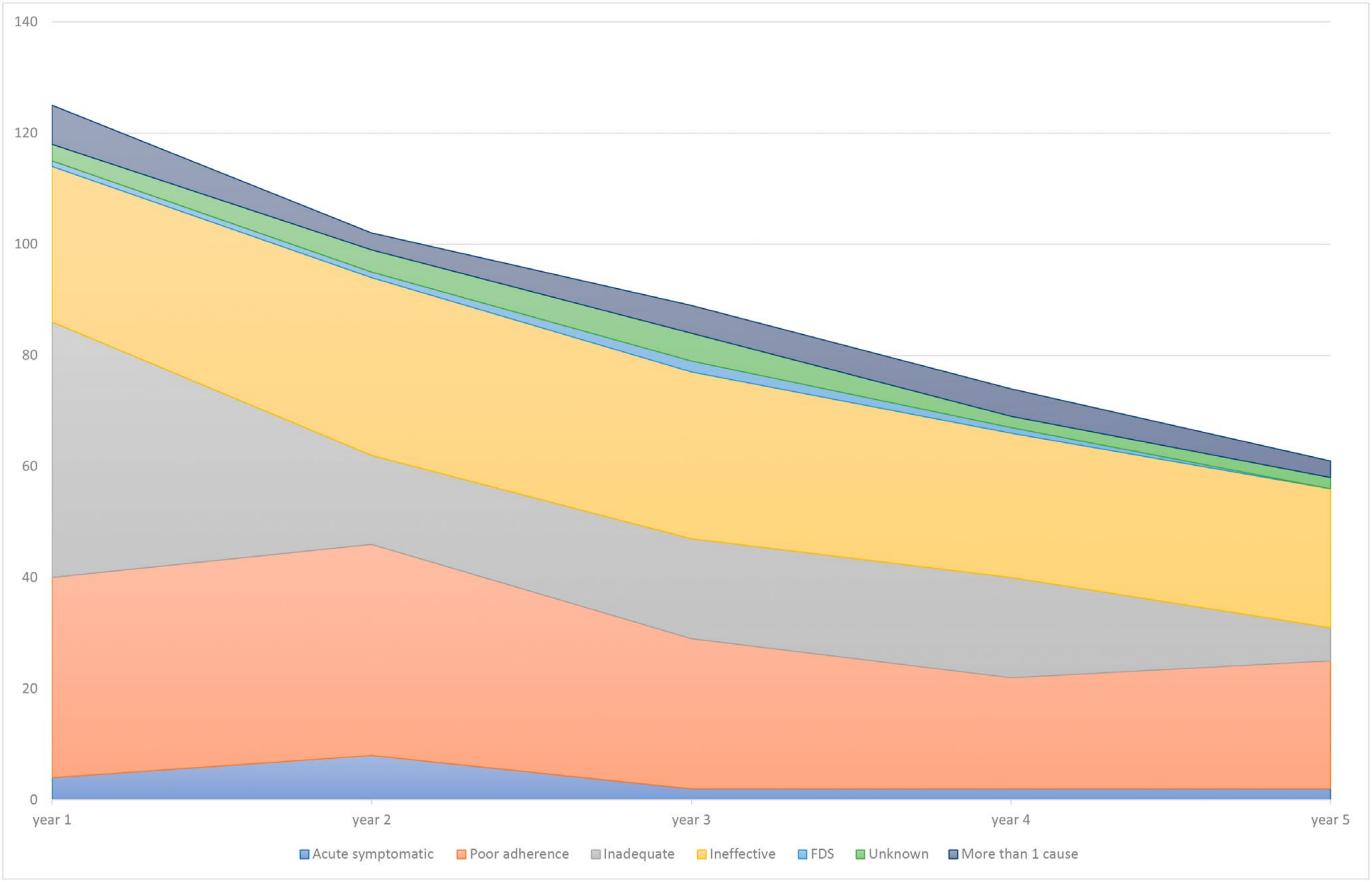
Distribution of relapse causes over 5 years of follow‐up. FDS, functional/dissociative seizures.

During the 5‐year follow‐up, 76 patients experienced relapses due to poor adherence, 77 due to inadequate treatment, and 57 due to treatment ineffectiveness; 16 had acute symptomatic seizures, and FDS occurred in seven subjects. In 60 patients, more than one cause was identified over the years. The mixed logistic regression showed a significant interaction between causes and years (χ^2^ [24] = 98.12, *p* < .001). The causes and annual distribution of relapses are showed in Figure 2.

The proportion of poor adherence remained stable throughout the 5 years and was the cause of relapses in 36 patients in the first year (29% of those who relapsed that year), 38 in the second year (37%), 27 in the third year (30%), 20 in the fourth year (27%), and 23 in the fifth year (38%; Table 2). In the post hoc analysis, no significant differences were found across the years. A mixed logistic regression examining factors associated with poor treatment adherence (epilepsy type and etiology, age, sex, seizure type, and seizure timing such as during wakefulness or sleep) revealed that patients with poor adherence were significantly younger (mean = 40.2 years ±18.7) than those who had never experienced a relapse for this reason (mean = 49.6 years ±20; χ^2^ [1] = 10.38, *p* = .001). Prior probable seizures were noted more frequently in patients who presented poor drug adherence (*p* = .001).

**TABLE 2:** Causes of relapse: Number of patients and percentages for each cause and each year.

Cause	Year 1, *n* (%)	Year 2, *n* (%)	Year 3, *n* (%)	Year 4, *n* (%)	Year 5, *n* (%)	*p* (years 1–Y5)
Total patients with relapses	125	102	89	74	61	<.001
Poor adherence	36 (29)	38 (37)	27 (30)	20 (27)	23 (38)	.86
Inadequate treatment	46 (37)	16 (16)	18 (20)	18 (24)	6 (10)	<.001
Ineffectiveness of treatment	28 (22)	32 (31)	30 (34)	26 (35)	25 (41)	.99
Acute symptomatic seizures	4 (3)	8 (8)	2 (2)	2 (3)	2 (3)	.99
FDS	1 (1)	1 (1)	2 (2)	1 (1)	0 (0)	.99
More than one cause suspected	7 (6)	3 (3)	5 (6)	5 (7)	3 (5)	.99
Unknown	3 (2)	4 (4)	5 (6)	2 (3)	2 (3)	.99

*Note*: Details on multiple causes are available in Tables S1–S4.

Abbreviation: FDS, functional/dissociative seizures.

All relapses attributable to inadequate treatment were due to subtherapeutic dosing; no cases were identified in which the prescribed medication was unsuitable for the patient's type of epilepsy or had a significant interaction with another drug. Inadequate treatment accounted for relapses in 46 patients (37% of those who relapsed that year) in the first year, 16 (16%) in the second year, 18 (20%) in the third year, 18 (24%) in the fourth year, and six (10%) in the fifth year (Table 2). The post hoc analysis revealed a significant decrease from the first year compared to subsequent years: year 2 (*Z* = 4.07, *p* < .001), year 3 (*Z* = 3.80, *p* = .001), year 4 (*Z* = 3.80, *p* = .001), and year 5 (*Z* = 5.12, *p* < .001). No significant differences were observed between years 2–5.

The number of patients with relapses due to treatment ineffectiveness remained grossly stable over the years but due to decreasing numbers of relapses, the relative proportion increased from 22.4% in the first year to 41% in the fifth year. Post hoc analysis did not find significant differences across the years.

ASyS, new onset FDS, and relapses occurring in unknown circumstances were rare, accounting for less than 10% of patients relapses each year, with no significant differences during follow up, although the small sample size in these groups does not allow for a reliable or generalizable analysis.

### 3.3 Evolution of DRE

Among the 57 patients who experienced seizure relapses due to treatment ineffectiveness (17% of the entire population of 330), 22 of 57 (39%) achieved seizure freedom with a second ASM; three (5%) were classified as having undefined drug responsiveness, as they did not undertake a second ASM trial. Thirty‐two (56%) were classified as DRE according to the ILAE classification. However, among these patients, by the end of the follow‐up period, six of 32 (19%) achieved seizure freedom with a third ASM and three (9%) following epilepsy surgery, leaving 23 of 32 (72%) with active epilepsy that was resistant after ≥2 drugs at the end of the 5‐year follow‐up. This corresponds to an ultimate rate of patients of 7% (23/330) not achieving sustained seizure freedom during the first 5 years (or 26/330 [8%] if the three surgical cases are added to the DRE number). The absolute number and proportion of patients meeting DRE criteria each year increased significantly: 7% (9/125) in the first year, 19% (19/102) in the second year, 24% (21/88) in the third year, 27% (20/74) in the fourth year, and 38% (23/61) in the fifth year (*Q* = 17.5, *df* = 4, *p* = .0015), mainly due to a sharp increase starting with the second year and smaller, nonsignificant increases thereafter (comparison between year 2–5, *Q* = 7.5, *df* = 3, *p* = .057). Given the limited number of patients meeting criteria for DRE at the end of follow‐up (*n* = 23), the study was not adequately powered to perform a reliable multivariable analysis of clinical predictors of drug resistance.

### 3.4 Seizure type and relapse causes

The distribution of seizure types at the time of relapse (GTCS vs. non‐GTCS) was evaluated across different relapse etiologies over the 5‐year follow‐up period (Table S3). We used a mixed logistic regression to assess the association between seizure type at relapse and relapse cause over time, including an interaction term between seizure type and relapse cause. The dependent variable was the presence or absence of seizures, with seizure type, relapse cause, and year as independent variables, as well as age, sex, and epilepsy type (focal vs. idiopathic/genetic generalized epilepsy).

Patients experiencing relapses due to poor adherence exhibited a significantly higher proportion of GTCS across all years (*p* < .001). In contrast, relapses attributed to ineffective treatment involved predominantly non‐GTCS (*p* < .001). There were no significant differences in demographic characteristics, including age, sex, or epilepsy type. We did not find a relationship of seizure type with the other relapse causes.

## 4 DISCUSSION

Seizure recurrence is a common event in patients with treated epilepsy, and drug unresponsiveness is a relevant but not exclusive cause. In our study, this was true for approximately one third of patients (31%). A better understanding of alternative factors, such as poor adherence or suboptimal treatment, could help reduce unnecessary escalation to polytherapy. Other relevant findings from our retrospective study include the following: (1) the overall number of relapses tends to decrease over the first 5 years; (2) patients who experience relapses at the beginning of the illness due to poor adherence also show this in the further course of the follow‐up; (3) inadequate treatment (too low dosage) is the predominant relapse cause during the first year; (4) relapses due to DRE were already seen in the first year, but the percentage increases sharply in the second year; and (5) GTCS relapses are mainly associated with poor adherence.

In another study of 1098 patients followed at a single center, 31% and 39% experienced a relapse after 2 and 5 years, respectively.^3^ Similar numbers were found in an initial study from the same center, published 12 years earlier.^21^ This compares favorably to the 31% relapse rate after 2 years in our study; however, after 5 years, we observed only 18%. This difference may be explained by the smaller catchment area of the University Hospital of Geneva or stronger ties to the Epilepsy Center. For example, patients often re‐present to the ED or contact the Epilepsy Center after a relapse and typically have a brief discussion with the EEG nurse or physician and, if deemed appropriate, a follow‐up EEG is performed the following day, allowing for an initial assessment of the most likely cause and the need for a more thorough follow‐up examination.

### 4.1 Poor adherence

A significant number of patients with poor treatment adherence were identified (27%–38% of relapses during 5 years). This number decreased only slightly over the years and without significant difference across the years of follow‐up. Furthermore, nonadherent patients were significantly younger. Our observations are consistent with existing literature describing poor treatment adherence as a major cause of pseudoresistance in epilepsy, with reported rates of approximately 40%.^22^, ^23^, ^24^ Factors associated with poor adherence include suboptimal clinician–patient communication, reduced frequency of clinical visits, and side effects of ASM.^23^, ^25^, ^26^ Demographic and socioeconomic factors, such as younger age, male gender, lower socioeconomic status, irregular insurance status, and limited education, have also been identified as contributing factors.^27^, ^28^, ^29^ These elements are relatively constant over time, which may explain the stable rates of nonadherence. Although we could not confirm a male predominance, our data suggest that patients often had a history of noninvestigated seizurelike events that were trivialized or neglected. Previous studies have shown that patients with a history of prior uninvestigated seizures have a higher risk of seizure recurrence,^30^ in which poor adherence may play a contributing role. Diagnosing and addressing compliance is critical, as it is associated with increased mortality and significantly impaired quality of life.^23^, ^31^ Successful interventions aimed at improving adherence include prescribing once‐daily ASMs, using newer generation ASMs with fewer adverse effects, regularly monitoring ASM blood levels, and implementing behavioral strategies.^32^, ^33^, ^34^

### 4.2 Inadequate treatment

The proportion of relapses attributable to inadequate treatment declined significantly over time, accounting for 37% of relapses in the first year and only 10% in the fifth year. Multiple reasons were identified. Elderly patients (>60 years), who made up 36% of the cohort, often received lower ASM dosages due to concerns about side effects; in other cases, treatment was deliberately started at reduced doses to improve tolerability (and continued compliance). In some cases, slow dose escalation (e.g., with lamotrigine) was mandatory and led to relapses before therapeutic levels were reached. No cases of inappropriate drug choice were identified, likely due to the predominant use of broad‐spectrum ASMs.

Although this cause decreased over time as expected, 10% of relapses in the fifth year were due to inadequate treatment (most often due to low seizure frequency and less severe clinical presentation, as well as side effects with higher dosages). Several previous studies have highlighted inadequate treatment as a significant contributor to seizure persistence in NOE.^24^, ^35^, ^36^ Our results confirm these observations but add the insight that inadequate treatment plays a significant role particularly in the first year after initiating therapy. This relapse cause decreases thereafter, as adjustments made during subsequent checkups address these deficiencies. Regular medication monitoring helps to identify this otherwise preventable cause of relapse.

### 4.3 Treatment ineffectiveness and DRE

Treatment ineffectiveness was observed already during the first year in some patients but increased significantly starting at year 2. Interestingly, the numbers remained relatively stable over the years (25–32), although the relative proportion increased due to the reduction of patients experiencing a relapse across the years (22% in the first year to 41% in the last year). Studies have reported a wide range of relapse rates after the first ASM trial, from 15% to 76%.^3^, ^36^, ^37^, ^38^ However, most of these studies did not specifically address the various causes of relapse. When other causes are actively researched, treatment ineffectiveness rates are below 20%,^35^, ^36^ which corroborates our findings (10%–13% of the total population in years 2–5).

Of all patients who did not respond to two ASMs, six of 330 (2%) achieved seizure control with a third ASM, and three of 330 patients (1%) underwent epilepsy surgery. Thus, at the end of the observation period, only 7% of our entire NOE cohort exhibited drug‐induced seizure resistance (DRE), or 8% if the surgical cases are added. Our results are comparable to, although somewhat lower than, those of other studies reporting early DRE rates between 12% and 15%,^39^, ^40^, ^41^ and lower than the 30%–40% frequently reported in studies over longer follow‐ups.^42^, ^43^ A lack of response to the first medication is a significant risk factor for DRE and can be noted already during the first 2 years.^42^ Therefore, the possibility of surgical treatment should be discussed at the time of diagnosis of focal epilepsy and/or failure of the first ASM, especially because early surgical treatment is associated with a higher probability of seizure control.^44^, ^45^ We cannot exclude that the high proportion of patients lost to follow‐up after the initial evaluation may have affected the estimation of the final rate of drug resistance. However, a systematic selection bias with an overrepresentation of milder cases is less likely, given our center's specialization in DRE and epilepsy surgery.

### 4.4 Association between seizure type at relapse and relapse cause

We observed a significant association between relapse due to poor adherence and GTCS, whereas relapses attributed to underdosing or drug ineffectiveness were predominantly associated with nongeneralized seizures. One possible pathophysiological explanation is that even with low ASM blood levels, ASM may still exert some efficacy in controlling the spread of epileptic activity and potentially reduce the development of GTCS.^46^ Also, abruptly stopping ASM, as is often the case in fluctuating adherence, can lead to withdrawal effects and increase the risk of GTCS. Given the strong association of GTCS and risk of sudden unexpected death in epilepsy,^47^ this finding underscores the importance of addressing poor treatment adherence.

### 4.5 Limitations

This study has several limitations inherent to its retrospective, single‐center design. The reliance on patient reports and existing medical records may have affected the accuracy of the information collected. Being conducted at a single center in a country with an affluent health care system, the findings of our study may not be generalizable to other populations with different demographics, health care systems with more limited access to expert care, or different clinical practices. Another significant limitation is the substantial proportion of patients (410/740) lost to follow‐up, which may introduce selection bias. Recontacting these patients was not feasible, as many were first evaluated 15–20 years ago, and updated contact information was unavailable. This high attrition rate may partly reflect the demographic and geographic characteristics of Geneva, including a highly mobile international population and cross‐border health care with France. Although a bias related to disease severity cannot be excluded, our center's specialization in DRE and epilepsy surgery makes a systematic overrepresentation of milder cases less likely. Despite the modified definition of epilepsy by the ILAE, a recent meta‐analysis on the prognostic relevance of epileptiform abnormalities on EEG after the first event, and the observation that prompt ASM introduction improves outcome, many neurologists and epileptologists prefer to wait for a second event before considering ASM.^48^ The sample size for subgroup analysis such as patients with DRE may be insufficient to detect statistically significant associations with clinical variables. Assessing patient adherence to ASM regimens is complex; relying solely on therapeutic drug monitoring and reports may not fully capture the adherence behavior, potentially leading to misclassification. Finally, it cannot be ruled out that the 330 patients treated at our center for 5 years are not representative of all NOE patients.

## 5 CONCLUSIONS

Despite these limitations, we believe that our detailed analysis of NOE patients with 5‐year follow‐up helps us better understand why and when patients relapse. Although ineffective treatment emerges as the primary cause of seizure persistence after 2 years and beyond, inadequate treatment (too low dose) significantly contributes to relapses in the first year, whereas poor adherence remains a consistent cause throughout the first 5 years.

Our findings underscore the need for evaluation after each relapse to adequately address the underlying cause with appropriate adjustments, new drug therapies, or additional educational interventions. Optimizing ASM or the timing of epilepsy surgery, monitoring adherence, and individualized follow‐up strategies are crucial for successful patient care. Adequately addressing modifiable factors reduces the risk of misclassifying patients as drug‐resistant and improves long‐term outcomes. Treatment by specialized centers or neurologists^49^ is associated with lower mortality and possibly also a lower risk of relapse. Future research should focus on developing early, individualized interventions and protocols, to improve treatment adherence and optimize strategies for initiating drug and surgical treatment. The goal is to further improve long‐term seizure control in patients with newly diagnosed epilepsy.

## AUTHOR CONTRIBUTIONS

**Cecilia Catania:** Study design; data collection and interpretation; manuscript drafting. **Eric Menetre:** Statistical analysis; data interpretation; manuscript revision. **Aikaterini Fitsiori, Felix Tobias Kurz, Roland Wiest:** Data interpretation; manuscript revision. **Gabriela Moreno Legast, Serge Vulliemoz, Mathieu Genoud, Pierre Mégevand, Fabienne Picard:** Data collection and interpretation; manuscript revision. **Sandor Beniczky:** Study design; data interpretation; manuscript revision. **Margitta Seeck:** Study supervision; study design; data interpretation; manuscript drafting and revision.

## FUNDING INFORMATION

This work was supported by SNSF 163398 (M.S.).

## CONFLICT OF INTEREST STATEMENT

E.M. is a shareholder in dEEGtal Insight. M.S. is a shareholder in dEEgital Insight and Epilog. S.V. is a shareholder in Epilog. None of the other authors has any conflict of interest to disclose. We confirm that we have read the Journal's position on issues involved in ethical publication and affirm that this report is consistent with those guidelines.

## Supporting information


Tables S1–S4.

## Data Availability

Deidentified data are available upon reasonable request.
