# Supplementary material for: Seizure relapse in new onset epilepsy: It is not always drug resistance
Source: Epilepsia. 2026 Jun 11;67(8):4183–93. doi: 10.1002/epi.70298 (PMC13525564; doi:10.1002/epi.70298)
Supplement: Supplementary file 1 [file EPI-67-4183-s001.docx]

|  | year 1 | year 2 | year 3 | year 4 | year 5 |
| --- | --- | --- | --- | --- | --- |
| Acute Symptomatic | 3 | 6 | 3 | 5 | 4 |
| Poor adherence | 55 | 68 | 33 | 28 | 56 |
| Inadequate | 53 | 12 | 10 | 13 | 2 |
| Ineffective | 29 | 35 | 34 | 24 | 16 |
| FDS | 0 | 0 | 0 | 0 | 0 |
| unknown | 2 | 2 | 3 | 2 | 1 |

eTable 1. n° of GTC and causes per year

| year 1 |  | Acute Symptomatic | Poor adherence | Inadequate | Ineffective | FDS | unknown |
| --- | --- | --- | --- | --- | --- | --- | --- |
|  | 1/year | 1 | 6 | 13 | 8 | 0 | 1 |
|  | >1/year | 0 | 4 | 10 | 10 | 0 | 0 |
|  | monthly | 0 | 0 | 2 | 8 | 1 | 0 |
|  | weekly | 0 | 2 | 1 | 1 | 0 | 0 |
|  | daily | 0 | 0 | 1 | 0 | 0 | 0 |
| year 2 |  | Acute Symptomatic | Poor adherence | Inadequate | Ineffective | FDS | unknown |
|  | 1/year | 2 | 4 | 4 | 4 | 0 | 2 |
|  | >1/year | 1 | 5 | 6 | 9 | 2 | 0 |
|  | monthly | 0 | 3 | 1 | 9 | 0 | 0 |
|  | weekly | 0 | 3 | 0 | 3 | 1 | 0 |
|  | daily | 0 | 0 | 0 | 0 | 0 | 0 |
| year 3 |  | Acute Symptomatic | Poor adherence | Inadequate | Ineffective | FDS | unknown |
|  | 1/year | 0 | 4 | 4 | 3 | 2 | 2 |
|  | >1/year | 0 | 4 | 4 | 11 | 1 | 0 |
|  | monthly | 0 | 5 | 2 | 6 | 1 | 0 |
|  | weekly | 0 | 2 | 0 | 4 | 1 | 0 |
|  | daily | 0 | 0 | 0 | 0 | 0 | 0 |
| year 4 |  | Acute Symptomatic | Poor adherence | Inadequate | Ineffective | FDS | unknown |
|  | 1/year | 0 | 1 | 6 | 3 | 0 | 0 |
|  | >1/year | 1 | 3 | 4 | 10 | 3 | 0 |
|  | monthly | 0 | 2 | 3 | 7 | 1 | 0 |
|  | weekly | 0 | 3 | 2 | 3 | 1 | 0 |
|  | daily | 0 | 0 | 0 | 0 | 0 | 0 |
| year 5 |  | Acute Symptomatic | Poor adherence | Inadequate | Ineffective | FDS | unknown |
|  | 1/year | 1 | 2 | 0 | 4 | 0 | 0 |
|  | >1/year | 0 | 1 | 3 | 6 | 1 | 1 |
|  | monthly | 0 | 4 | 1 | 6 | 1 | 0 |
|  | weekly | 0 | 1 | 1 | 3 | 1 | 0 |
|  | daily | 0 | 0 | 0 | 0 | 0 | 0 |

eTable 2. Frequency of non GTC seizure and causes per year

| N° patients with GTCS | |  |  |  |  |  |
| --- | --- | --- | --- | --- | --- | --- |
|  | Acute Symptomatic | Poor adherence | Inadequate | Ineffective | FDS | unknown |
| year 1 | 3 | 28 | 30 | 17 | 0 | 2 |
| year 2 | 5 | 31 | 9 | 18 | 0 | 2 |
| year 3 | 2 | 21 | 8 | 13 | 0 | 4 |
| year 4 | 1 | 16 | 10 | 9 | 0 | 2 |
| year 5 | 2 | 20 | 2 | 8 | 0 | 1 |
| n patients with n-GTC | |  |  |  |  |  |
| year 1 | 1 | 12 | 27 | 27 | 1 | 1 |
| year 2 | 2 | 15 | 11 | 25 | 3 | 2 |
| year 3 | 0 | 15 | 10 | 24 | 5 | 2 |
| year 4 | 1 | 9 | 15 | 23 | 5 | 0 |
| year 5 | 1 | 8 | 5 | 24 | 3 | 1 |

eTable 3. Seizure type according to seizure etiology over the years

|  | year 1 | | year 2 | | year 3 | | year 4 | | year 5 | |
| --- | --- | --- | --- | --- | --- | --- | --- | --- | --- | --- |
| N° patients | 7 |  | 3 |  | 5 |  | 5 |  | 3 |  |
|  | 5 | Inadequate + ineffective | 1 | FDS + poor adherence | 2 | FDS + poor adherence | 3 | FDS + poor adherence | 2 | FDS + poor adherence |
|  | 1 | Poor adherence + inadequate | 1 | FDS + inadequate | 1 | FDS + inadequate | 1 | FDS + inadequate | 1 | FDS + inadequate |
|  | 1 | Poor adherence + ineffective | 1 | Poor adherence + ineffective | 2 | Poor adherence + ineffective | 1 | Poor adherence + ineffective |  |  |

eTable 4. Specific causes of relapse in patients having multiple relapse circumstances in the same year.
